# Concomitant Myocardial Infarction and Stroke Managed With a Unique Approach: A Case Report and Review of Literature

**DOI:** 10.7759/cureus.78073

**Published:** 2025-01-27

**Authors:** Hitesh Gurjar, Himani Singh

**Affiliations:** 1 Department of Medicine, University of Arizona College of Medicine, Tucson, USA; 2 Department of Cardiac Sciences, Livasa (Formerly Ivy) Hospital, Nawanshahr, IND; 3 Department of Radiology, Livasa (Formerly Ivy) Hospital, Nawanshahr, IND

**Keywords:** acute cardio-cerebral infarction, acute ischemic stroke (ais), acute myocardial infarction (ami), acute myocardio-cerebral infarction, acute myocardio-cerebral infarction (amci), catheter-directed thrombolysis, cerebral intraarterial thrombolysis, hyperacute cardio-cerebral infarction, intracoronary thrombolysis, simultaneous acute myocardial infarction and acute ischemic stroke

## Abstract

A 71-year-old gentleman presented with concomitant acute myocardial-cerebral infarction (AMCI). The patient was treated with a novel endovascular approach of simultaneous intracoronary (IC) and cerebral intra-arterial (IA) thrombolysis. The concomitant occurrence of acute myocardial infarction (AMI) and acute ischemic stroke (AIS) is a rare and challenging clinical scenario with a lack of established definitions and treatment guidelines. The preferred treatment approach of combined mechanical thrombectomy (MT) and percutaneous coronary intervention (PCI) is not widely available, hence necessitating the need to explore further practical and feasible treatment options.

## Introduction

Concomitant occurrence of AMI and AIS is a rare phenomenon. After the publication of the case report by Omar et al., most of the prospective data was via case reports only [1-3]. Most of the evidence is generated from retrospective data, which shows incidence varying from 0.009% to 1.6 % [4-6]. After an episode of AMI, the risk of stroke is 1.4-3.7%, with the highest risk being within the first four weeks but persisting up to three months [7]. Similarly, a one-year risk of AMI after an episode of AIS or transient ischemic attack (TIA) remains around 2% [8]. Few studies have excluded patients who developed stroke during AMI to exclude procedure-related deaths.

In contrast, others include only procedural-related AIS after AMI, thus, in turn, limiting accurate estimation of the incidence of concomitant AMI and AIS [6,7]. The outcomes are worse for AMCI than for patients presenting with either AMI or AIS [9]. The mortality rates are estimated to be 20-30% at the end of one year [10]. Management of such cases is especially challenging due to the lack of wider availability of MT and the paucity of literature to guide treatment choices. Treatment options can include pharmacological thrombolysis, either systemic (full dose vs reduced dose) or catheter-directed versus interventional, combining percutaneous coronary interventions (PCI) with cerebral MT. Despite the better outcomes shown by the invasive approach, PCI is only performed in less than 2% of cases of AMCI [4]. The combined use of MT and PCI is even rarer [2,11].

Below, we describe a case where, due to the non-availability of MT, we used the intracoronary (IC) and cerebral intra-arterial (IA) approach with catheter-directed delivery of reduced-dose thrombolytics with a successful outcome. To the best of our knowledge, this is the first instance where such an approach has been successfully employed in this subset of patients. We propose that concomitance should be determined when patients have both events within 24 hours of each other, and both should be present at the time of presentation. The reason for considering a 24-hour period is due to evolving time windows for stroke interventions, which can now be offered up to 24 hours [12]. Similarly, PCI is usually beneficial before the 24-hour window, preferably less than 12 hours [13].

## Case presentation

A 71-year-old right-handed male patient was brought in by his spouse to the emergency room (ER) for right-sided weakness and chest discomfort for 4.5 hours duration. His past medical history was significant for hypertension. He did not have diabetes mellitus, hyperlipidemia, or any other systemic disease. He did not smoke cigarettes, consume alcohol, or use recreational drugs. He did not have any history of febrile illness. He was taking amlodipine for his hypertension.

**Figure 1:**
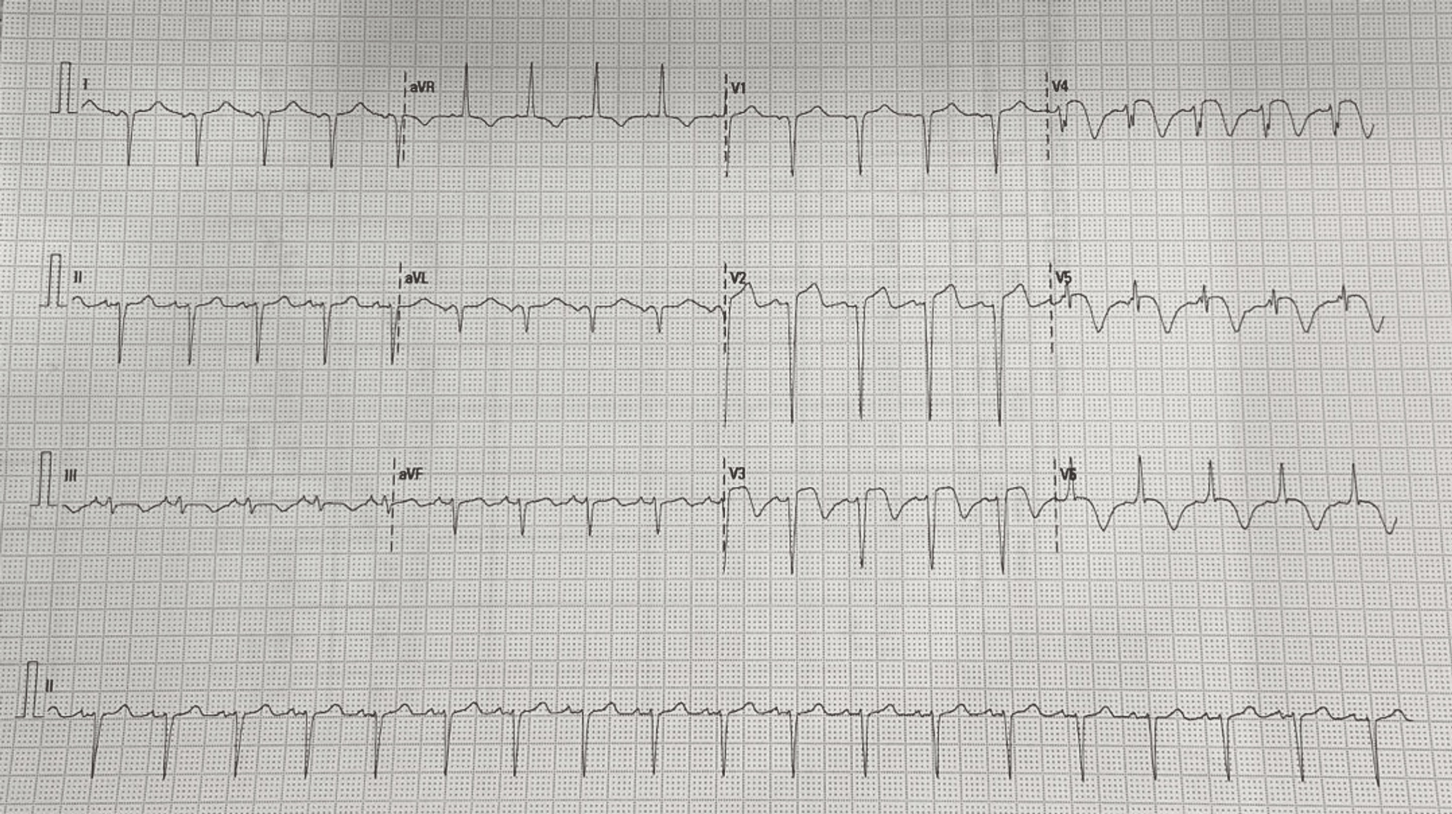
Electrocardiogram showing evolved acute anterior wall myocardial infarction with ST elevation in precordial leads V2 to V5 with Q-waves.

In the ER, the patient was noted to be aphasic and dysarthric with right-sided upper and lower limb power of 0/5. The National Institute of Health Stroke Scale (NIHSS) score was 13. The patient had a temperature of 98.6°F, a blood pressure of 160/86 mmHg, a heart rate of 86 beats per minute, a respiratory rate of 16 breaths per minute, an oxygen saturation of 100% while breathing ambient air, and a random blood sugar level of 108 mg/dL. The chest was clear to auscultation, and the rest of the exam was unremarkable. The electrocardiogram showed extensive ST-segment elevation in leads V2 to V5, I, and aVL (Figure 1).

**Figure 2:**
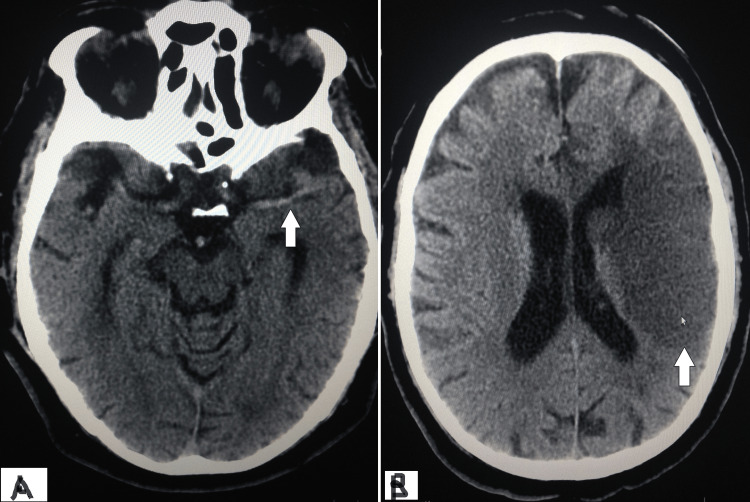
(A) Initial computed tomography (CT) of head showing left side middle cerebral artery as shown with white arrow, dense MCA sign (B) CT head, after 12 hours, shows fully evolved left side MCA territory infarct as depicted by white solid arrow.

He underwent urgent non-contrast computed tomography (NCCT) of the head, which showed a dense middle cerebral artery (MCA) sign and no evidence of bleed (Figure 2A). A loading dose of 325 mg of aspirin, 300 mg of clopidogrel, and 80 mg of atorvastatin was given. A bedside echocardiogram was done, which showed hypokinesia in the left anterior descending coronary artery (LAD) territory and visually estimated ejection fraction of 35 to 40%. Since he presented to the hospital after 4.5 hours, hence intravenous alteplase (tPA) was not considered. He was emergently taken to the catheterization lab for a coronary angiogram (CAG) with the initial intention of revascularization.

**Figure 3:**
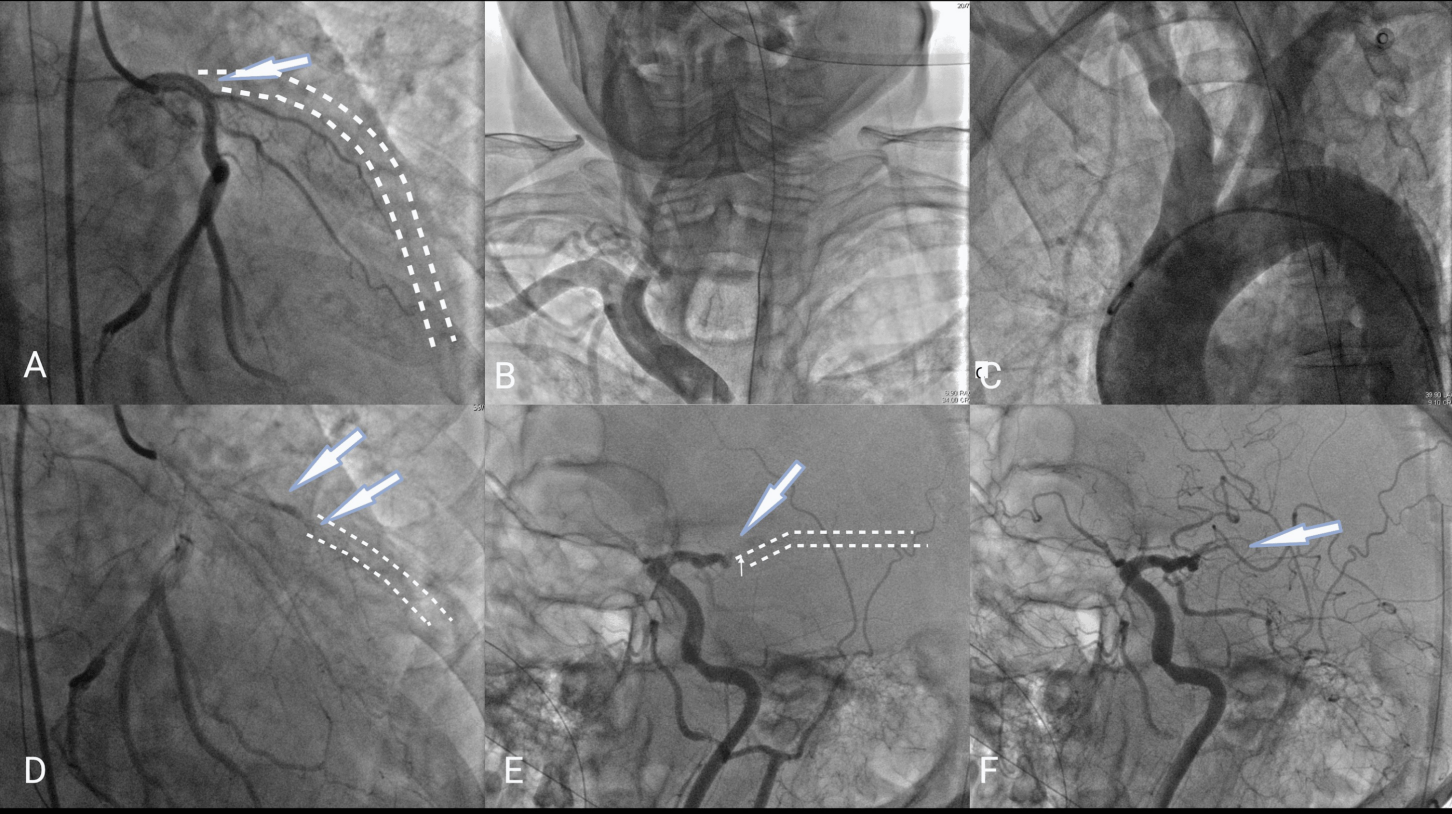
Panel showing coronary and cerebral angiogram images before and after intra-coronary and intra-cerebral thrombolysis with alteplase. (A) The angiogram shows a totally occluded LAD in the RAO caudal view. LCX is seen to be normally filling. Broken lines and arrows show the expected path of LAD. (B) A carotid angiogram shows normal right-sided extra-cranial arteries (C) aortic arch aortogram with a 6f pigtail catheter showing a normal aortic arch and its branches. (D) Coronary angiogram images in RAO caudal view show improved filling in LAD after intra-coronary injection of alteplase. The broken lines show the part of LAD visualized in later parts of the cine. (E) The cerebral angiogram shows occluded left MCA after the M1 branch, and broken lines represent the expected path of MCA. (F) A cerebral angiogram after the intra-cerebral aliquots of alteplase showed improved filling of the left MCA territory appreciated with flow in multiple branches supplying the MCA territory. RAO: right anterior oblique; MCA: middle cerebral artery.

However, CAG revealed an ostial LAD thrombotic 100% flush cut-off appearance (Figure 3A). Left main (LM) coronary artery, right coronary artery (RCA), and left circumflex coronary artery (LCX) were normal. A cerebral angiogram showed complete occlusion of the M2 branch of the left MCA (Figure 3E). 

Rationale and clinical decision-making

He was considered for combined PCI with MT, but due to the non-availability of such a specialized center in the nearby region, it was deferred. Further plan was revised to intervene with the best available resources. Given the high risk of thrombus migration during PCI in cases of ostial flush cut-off LAD vessels, he was offered targeted IC thrombolysis. Since the patient had also received a full dose of heparin and an antiplatelet loading dose, he would have been at much higher risk of bleeding with conventional thrombolysis doses (which would have been 72 mg alteplase for an 80-kilogram weight patient). Hence, he was chosen for a lower dose of targeted thrombolysis. He was administered 25 milligrams of alteplase (tPA) via the intracoronary route and 20 mg (in 5 mg aliquots) to the left MCA via microcatheter. The patient was noted to have improved filling of the mid and distal parts of the LAD and improved flow trickling in MCA (Figures 3D, 3F). 

He was moved and further managed in the coronary care unit (CCU). The laboratory results reported hemoglobin of 12.7 gm/dL, white blood cell count of 12,100/mm^3^, platelet count of 245x10^9^/mm^3^, and serum creatinine of 0.9 mg/dL. CT head was repeated after 12 hours, which showed an evolved left MCA infarct without any bleeding (Figure 2B). The chest pain improved within 60 minutes after the procedure. He also started improving the strength of the right side of the body on the second day. His repeat echocardiogram revealed no mechanical complications, and LVEF was the same at 35-40%. He was discharged after five days with a power of 3/5 on the right side to a rehabilitation center. He was continued on aspirin, clopidogrel, metoprolol, lisinopril, and atorvastatin. At a follow-up of three months, he slowly improved with being able to ambulate with the help of a walker. 

## Discussion

**Figure 4:**
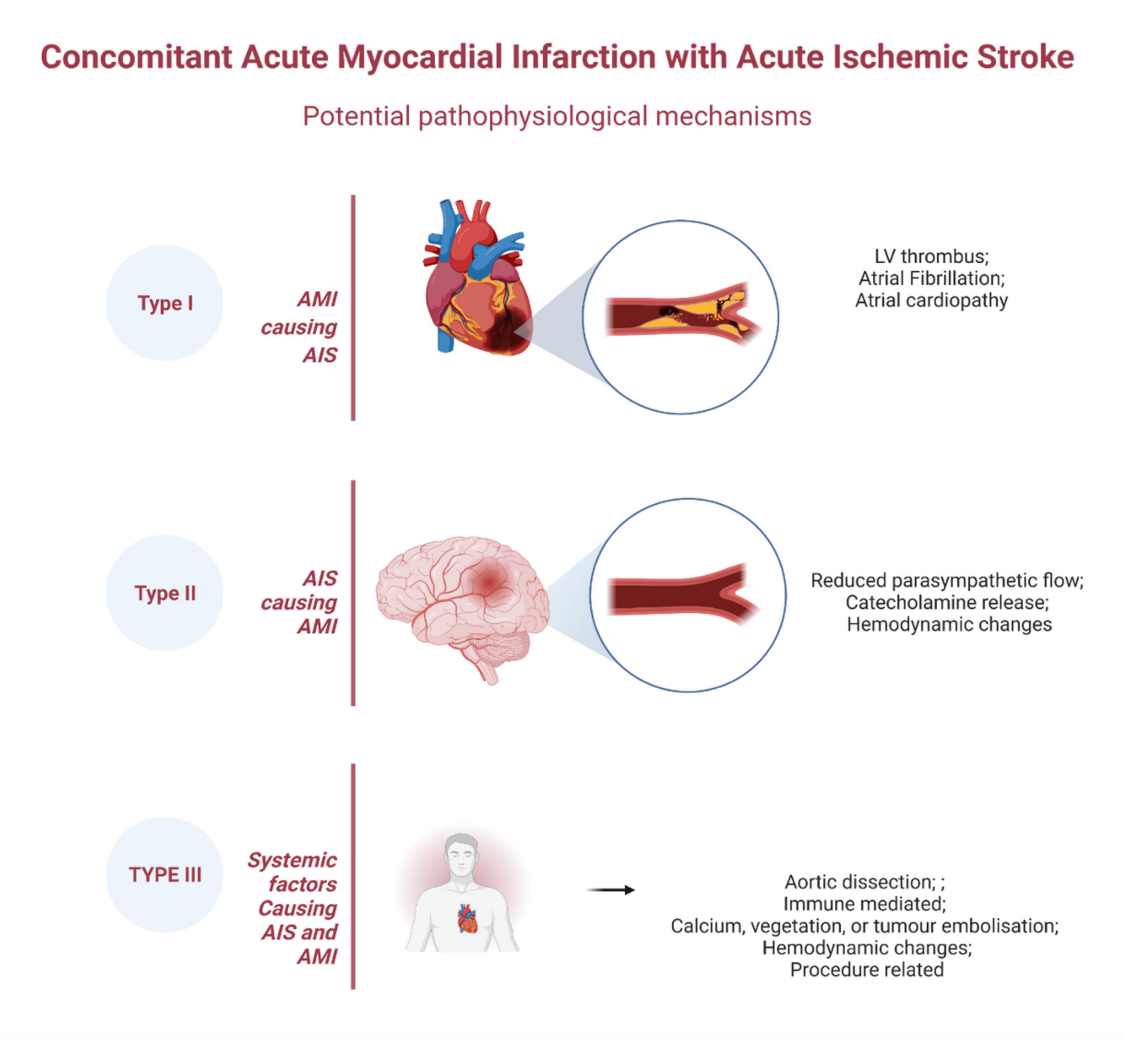
Diagram showing possible mechanisms causing the concomitant myocardial and cerebral involvement Created in BioRender. Gurjar, H. (2025) https://BioRender.com/d21v416

The concomitant occurrence of AMI and AIS is a rare but high-risk condition. Various factors have been proposed to explain the occurrence of AIS after AMI and vice versa. Factors causing AIS after AMI are predominantly linked to left ventricular (LV) thrombus embolization, atrial fibrillation (AF), or atrial cardiopathy [9,14,15]. Key factors contributing to AMI following an episode of AIS include acute blood pressure fluctuations, catecholamine release, and reduced parasympathetic activity [16,17]. Additionally, systemic factors such as accelerated hypertension, acute systemic hemodynamic changes, procoagulant states (e.g., post-COVID-19), and immune-mediated conditions like myocarditis and cerebritis can simultaneously impact both the heart and brain, leading to concurrent AIS and AMI. Embolization from sources upstream of the coronary and cerebral arteries, including the left atrium (LA), left atrial appendage (LAA), mitral valve (MV) apparatus, left ventricular (LV) cavity, aortic valve (AV), or the right heart via a patent foramen ovale, may involve thrombi, vegetations, calcium, or tumor fragments [9,14-18]. Similarly, aortic dissections involving the ascending aorta can extend to the coronary and carotid arteries, resulting in simultaneous AMI and AIS (Table 1, Figure 4) [19].

**Table 1:** Proposed types of AMCI and possible causes and mechanisms leading to the phenomenon. AMCI: acute myocardio-cerebral infarction.

Types	Description	Causes	Mechanisms
Type 1	AMI causing AIS (before any intervention)	LV thrombus, atrial fibrillation, or atriopathy, hemodynamic changes	Embolization of intracardiac thrombus, hemodynamic changes causing reduced cerebral perfusion and causing watershed area infarct
Type 2	AIS causing AMI (before any intervention)	Left parietal lobe involvement, right parietal lobe involvement, insular cortex involvement (demand ischemia or isolated troponin elevations are excluded)	Reduced parasympathetic outflow, increased sympathetic outflow, release of catecholamines causing myocardial infarction, blood pressure, and baroreceptor changes
Type 3	Systemic factors causing AMI and AIS	Systemic embolization causing both phenomena, systemic hemodynamic changes, aortic dissection, immune/inflammation-mediated injury due to infections, drugs, vaccines, or autoimmune conditions, procedure-related	Embolization of aortic or mitral valve calcium/tumor to both coronaries and cerebral circulation, left atrial or left atrial appendage thrombus or vegetation migration, extension of aortic dissection to carotids and coronaries, immune/Inflammation mediated myocarditis and cerebritis due to vaccine, infection, drugs, or autoimmune factors, procedure-related complications when intervening for either AMI or AIS

The presence of ST-segment elevation MI (STEMI), especially in anterior wall location and location of stroke, has been shown to affect outcomes and management in the literature. Location of infarct in the dominant left parietal lobe has been associated with increased cardiac death (adjusted HR of 4.45; 95% CI 1.83 to 10.83), with right parietal lobe infarction being associated with slightly lesser chances of cardiac death or MI (adjusted HR of 3.3; 95% CI, 1.45 to 7.51) [17]. Preclinical studies have also implicated left insular cortex infarction as being associated with decreased parasympathetic outflow, leading to cardiac effects [16]. The index patient had left MCA territory involvement with anterior wall STEMI, putting him at higher risk of complications and poorer prognosis.

Treatment options can be broadly divided into thrombolytic-based therapy and endovascular interventional approach. At centers where the capability of MT and PCI is available, combined treatment involving a multidisciplinary team approach is the preferred management strategy. However, the MT facility is not widely available compared to PCI facilities. Hence, consideration of alternate management strategies for such patients is needed. 

Thrombolysis is an established treatment modality for both AMI and AIS. Intravenous thrombolysis for AIS is recommended for up to 4.5 hours and for AMI up to 12 hours (24 hours if associated with cardiogenic shock) [20,21]. IC thrombolysis has been in use since 1983, when many of the initial historical PCIs included the delivery of IC thrombolytics [22]. However, with the development of stents, it lost favor. In certain situations, it remains a useful adjunct, such as in patients with large ectatic vessels with high thrombus burden, saphenous venous grafts, and cases where the harm of the procedure is deemed more than the benefit based on operator preference [23,24]. Recommended dosages have varied from 10 mg to 70 mg for alteplase given either as a bolus or via slow infusion. These dosages are significantly less when compared to systemic thrombolysis (<100 mg in AMI and <90 mg in AIS) [24-26]. Likewise, intra-arterial thrombolysis for stroke, although not approved in the United States, has been used as a treatment option for acute MCA stroke and can be instituted for up to six hours [27-29]. Since full-dose intravenous thrombolysis has been associated with increased complications of cardiac rupture and tamponade when used in patients with AIS who had STEMI (especially anterior wall STEMI within a preceding week), hence IC thrombolysis and intra-arterial (IA) thrombolysis can be an attractive alternative treatment option [30]. 

As of today, we face significant challenges and limitations in managing such patients. The lack of widespread availability of MT and PCI-capable centers and the expertise of physicians in managing such circumstances remain significant limiting factors that account for poor outcomes in such cases. We believe the wider availability of teams or individuals with experience in both coronary and cerebral interventions can be useful in managing such patients and can offer better clinical outcomes.

## Conclusions

Concomitant occurrence of AMI and AIS is a rare phenomenon with higher complication rates. However, it can be managed successfully if treatment is tailored according to patient-related, institution-related, and operator-related factors and preferences. While combined PCI and MT remains a preferred strategy, it is not widely available. Endovascular catheter-directed delivery of IC and IA thrombolysis can be a safe and effective way of managing a subset of such patients. An international consensus is required among experts to propose a case definition and effective management strategies. To the best of our knowledge, it is the first time this approach has been used. It is seemingly an effective and safe approach with the advantage of a reduced dose of thrombolytic agent and direct delivery at the site of the thrombus rather than systemically. However, further studies are needed before any recommendations can be made.
